# Bidirectional Association between Gastroesophageal Reflux Disease and Bipolar Disorder: A Systematic Review and Meta-analysis of Longitudinal Studies

**DOI:** 10.34172/mejdd.2025.411

**Published:** 2025-01-31

**Authors:** Rahma Nurita, Adit Faturohman, Febrina Mustika Santoso, Bianca Magdalena, Muhana Fawwazy Ilyas

**Affiliations:** ^1^Medical Doctor Program, Faculty of Medicine, Universitas Padjadjaran, Sumedang, West Java, Indonesia; ^2^Emergency Department, Ciremai Military Hospital, Cirebon, West Java, Indonesia; ^3^Medical Doctor Program, Faculty of Medicine, Universitas Hang Tuah, Surabaya, East Java, Indonesia; ^4^Medical Doctor Program, Faculty of Medicine, Universitas Diponegoro, Semarang, Central Java, Indonesia; ^5^Department of Anatomy and Embryology, Faculty of Medicine, Universitas Sebelas Maret, Surakarta, Indonesia; ^6^Department of Neurology, Faculty of Medicine, Universitas Sebelas Maret, Surakarta, Indonesia

**Keywords:** Bidirectional, Bipolar disorder, Comorbidity, Gastroesophageal reflux disease, Meta-analysis, Systematic review

## Abstract

**Background:**

Gastroesophageal reflux disease (GERD) and bipolar disorder impose substantial global burdens on individuals and healthcare systems. Previous studies suggest a bidirectional association between GERD and bipolar disorder. By searching and reviewing the results of existing studies, this systematic review and meta-analysis aims to review the two-way relationship between GERD and bipolar disorder.

**Methods:**

This study adhered to PRISMA Guidelines, including a comprehensive search of PubMed and Scopus for observational longitudinal studies. Quality (risk of bias) assessment employed the Newcastle-Ottawa Scale, and RevMan version 5.3 facilitated meta-analysis.

**Results:**

Five longitudinal studies (161888 patients) revealed a significant bidirectional link between GERD and bipolar disorder. Patients with GERD had a 2.29-fold higher risk of bipolar disorder (OR=2.29 [1.64, 3.21]; *P*<0.001), while individuals with bipolar disorder had a 2.80-fold higher risk of GERD (OR=2.80 [1.36, 5.76]; *P*=0.005). This study also identified independent risk factors, including sex, age under 60 years, and alcohol consumption disorders, influencing the occurrence of bipolar disorder in patients with GERD, as well as there is an influence of the number of psychoactive drugs in the occurrence of GERD in patients with bipolar disorder.

**Conclusion:**

These findings highlight a bidirectional relationship between GERD and bipolar disorder, emphasizing the necessity for integrated care models and personalized treatment plans. The results underscore the importance of considering both gastrointestinal and mental health aspects in managing these interconnected conditions.

## Introduction

 Gastroesophageal reflux disease (GERD) is prevalent in the world and causes a significant burden. Globally, the prevalence of GERD in 2019 was 9574.45 per 100 000 people, with an increasing trend of 0.105% per year since 1990.^1^ In Southeast Asia, the prevalence of GERD in 2005-2010 was 6.3%-18.3%.^2^ GERD is the most common outpatient diagnosis, with the number of visits reaching 9 million visits. Subsequently, proton pump inhibitor (PPI), as the main drug for GERD, covers 50% of the total prescriptions for all digestive diseases with an estimated health cost burden of up to 10 billion US dollars per year.^3,4^ A study by Meyiz et al showed that there was also moderate to severe impairment of quality of life in 62% of patients with GERD who received daily PPI therapy.^5^ Other studies showed poor quality of life scores in patients with GERD, especially related to psychological stress, which is a triggering factor for reflux symptoms.^6,7^

 Bipolar disorder is a common psychiatric disorder that may result in functional disability. The prevalence of bipolar disorder is 2.4%, with a prevalence of 0.6% for bipolar type I and 0.4% for bipolar type 2 worldwide.^8^ In 2015, the economic burden due to bipolar disorder reached 202 billion US dollars per year.^9^ Compared with the general population, patients with bipolar disorder have a worse quality of life score, which is mainly related to a decrease in social functioning and work productivity due to somatic disorders.^10,11^ Bipolar disorder has also been reported to reduce life expectancy by 9–20 years, which is associated with increased death rates from suicide.^12^ Subsequently, bipolar disorder also often causes functional gastrointestinal symptoms.^13^

 Several studies have shown a connection between GERD and psychiatric problems, one of which is bipolar disorder. A study by Lin and colleagues shows that GERD could increase the risk of developing bipolar disorder.^14^ This is also supported by Lee and co-workers, who found that GERD was a risk factor for psychological disorders, including bipolar disorder and depression.^15^ Meanwhile, another study by You et al showed contradictory findings, stating that there was no correlation between GERD and bipolar disorder.^16^ Several studies also show an increased risk of GERD in patients with bipolar disorder. Pan et al found that patients with bipolar disorder who used psychoactive drugs had a higher tendency to develop GERD.^17^ Avidan and colleagues also found that reflux symptoms occurred more frequently in patients with psychiatric disorders than in patients without psychiatric disorders.^18^ Another study by Rubenstein and co-workers also showed that GERD conditions were often associated with individuals who had other psychiatric disorders such as obsessive-compulsive disorder, depression, anxiety, phobic disorders, and paranoid ideation.^19^

 By searching and reviewing the results of existing studies, this systematic review and meta-analysis aimed to review the two-way relationship between GERD and bipolar disorder. Furthermore, the synthesis of evidence from various sources aimed to increase the optimization of clinical practice so that it can improve the quality of management and outcomes of patients with GERD or bipolar disorder.

## Materials And Methods

###  Search Strategy

 This is a systematic review and meta-analysis study based on the PRISMA 2020 Checklist.^20^ The search was conducted on February 28, 2024, in international databases, including Pubmed and Scopus. We formulated two PECO questions: (1) population: patients with GERD, exposure: GERD, comparison: none, outcome: risk of bipolar disorder; and (2) population: patients with bipolar disorder, exposure: bipolar disorder, comparison: none, outcome: risk of GERD. The keywords used were (“Gastroesophageal Reflux” OR “Esophageal Reflux” OR GERD OR “Gastric Acid Reflux” OR “Gastroesophageal Reflux” OR “Gastro-oesophageal Reflux”) AND (“Bipolar Disorder” OR “Bipolar Mood Disorder” OR “Manic Depression” OR “Manic Disorder” OR “Manic-Depressive” OR “Affective Psychosis”). We conducted a systematic search to collect relevant research, followed by a manual search of references cited in the included studies to prevent missing any relevant publications. The protocol of this study has been registered on the International Prospective Register for Systematic Review with the number CRD42024516587.

###  Eligibility Criteria

 An observational longitudinal study (cohort or case-control) evaluating the risk of bipolar disorder in patients with GERD and the risk of GERD in patients with bipolar disorder was included in the study. No restrictions were placed on the year and country of publication. Studies written in languages other than English or Bahasa Indonesia, and studies whose full text was not available were excluded.

###  Selection Process

 Four reviewers (RN, AF, FMS, BM) independently screened titles and abstracts to identify possibly suitable research during the selection process. Full-text papers were also evaluated for eligibility using the inclusion and exclusion criteria. Any disagreements among the reviewers were handled through mutual discussion and then with the consensus of the fifth reviewer (MFI).

###  Quality Assessment

 The Newcastle-Ottawa Scale (NOS) was used to evaluate the quality of cohort and case-control studies.^21^ The NOS has three primary components: selection of the study groups (0-4 points), comparability of cases and control studies (0-2 points) or cohorts, and ascertainment of exposure/outcome (0-3 points). Research with six or more points is deemed high-quality. Using this tool, four reviewers (RN, AF, FMS, BM) separately assessed the quality of the study, and any conflicts in the assessment among the reviewers were also handled through mutual discussion and then with the consensus of the fifth reviewer (MFI).

###  Data Analysis

 A qualitative and quantitative approach was used to identify data across studies. The data analysis in this systematic review consisted of summarizing the findings of the included studies using a narrative synthesis approach. The data extracted were the author, year of publication, study design, study location, sample size, and diagnostic method of GERD and bipolar disorder. Dichotomous data (frequencies and percentages) and numerical data (mean and standard deviation) were retrieved. Odds ratio (OR), hazard ratio (HR), and incidence rate ratio were reported with a 95% confidence interval. Any missing data found would be completed by contacting the study’s corresponding author. Four reviewers (RN, AF, FMS, BM) separately synthesized the study, and any conflicts in the extraction process among the reviewers were also handled through mutual discussion and then with the consensus of the fifth reviewer (MFI).

 A quantitative approach was performed using Review Manager 5.4. A forest plot estimating the OR was used to summarize the risk of the outcome. Statistical heterogeneity was evaluated by I^2^ statistics, with the cut-off values of 0%, 30%, 50%, or 75% for insignificant, moderate, substantial, or considerable heterogeneity, respectively. When the heterogeneity test results are insignificant or moderate, a fixed effect measure was used; while the heterogeneity is substantial or considerable, a random effect measure was used. Subsequently, funnel plots were used to identify publication bias.

## Results

###  Study Selection

**Figure 1:**
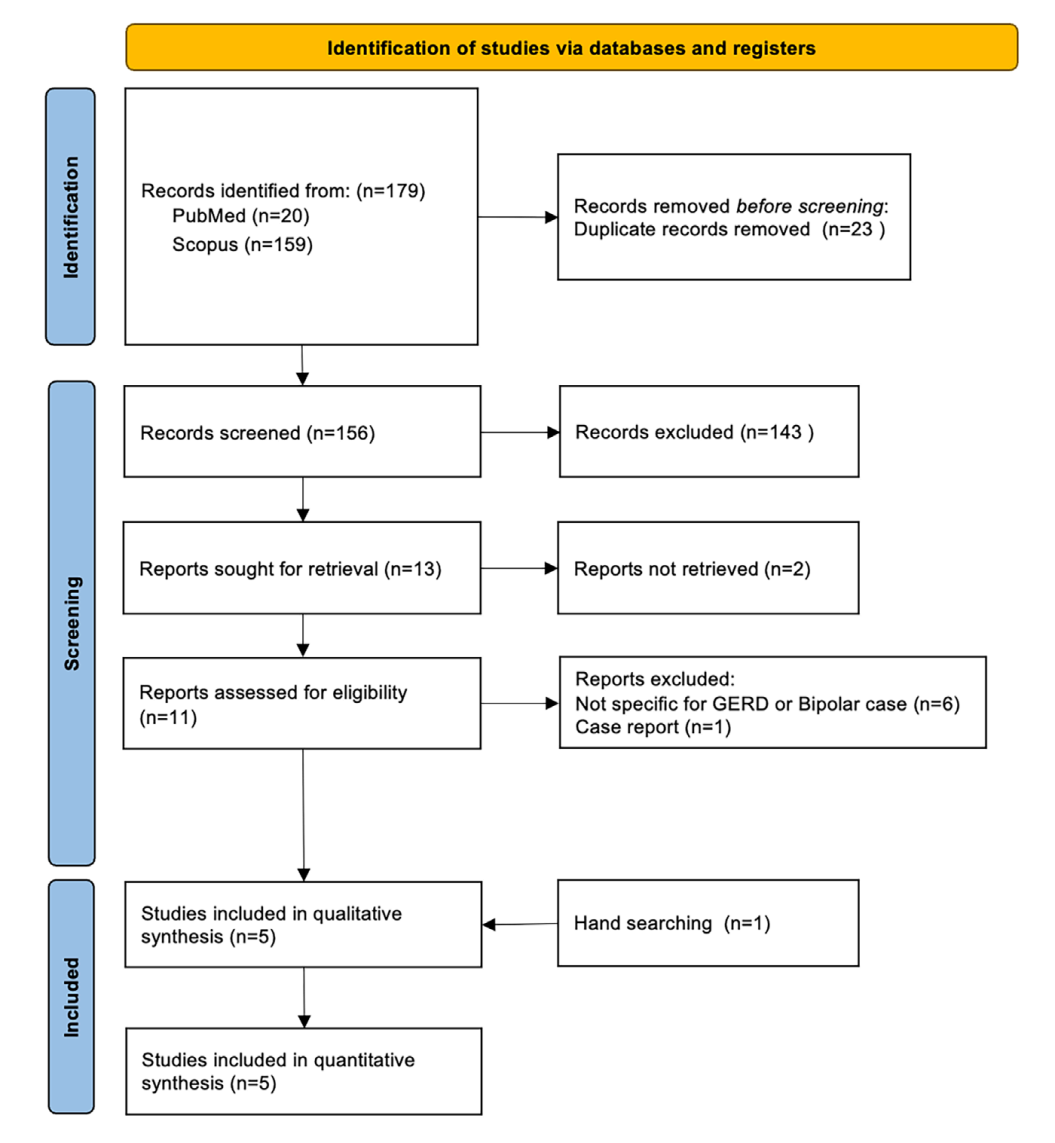
PRISMA diagram in the study selection process

 This review revealed a total of 179 studies, 20 of which came from Pubmed and 159 studies from Scopus in the first search process. Twenty-three duplicate studies were excluded from this study. Subsequently, a total of 156 studies underwent a screening process, with the results of 143 studies being excluded based on the title and abstract. A total of two studies did not have full-text papers available for examination. A total of 11 full-text papers were examined for study eligibility, with the results showing that six studies did not focus on discussing GERD and bipolar disease, and one study was a case report. There was one additional study based on the hand-searching process. Lastly, five articles were synthesized and assessed qualitatively and quantitatively. The process for collecting articles in this study is visualized in Figure 1.

###  Study Characteristics

 Based on the five studies included in this review, there were four retrospective cohort studies and one case-control study. Three studies were from Taiwan, while others came from the Republic of Korea and the United States. The number of samples from each study varied from 216 patients to 80 253 patients, with a total sample of 161 888 patients. The follow-up period was up to 10 years. All studies received a total of more than 6 points in the study quality assessment, so all studies used were good in study quality. The characteristics of the entire study are displayed in Table 1.

**Table 1:** Study characteristics

**Authors (year)**	**Study design**	**Country**	**Sample size**	**GERD diagnosis**	**Bipolar disorder diagnosis**	**Quality assessment score**
**Risk of bipolar disorder in patients with GERD**						
Lee et al (2018) ^15^	Retrospective cohort	Republic of Korea	Total: 19006 GERD: 9503 Non-GERD: 9503	KCD-6 codes K21.0 dan K21.9	KCD-6 codes F31.0-F31.9	8
Lin et al. (2014) ^14^	Retrospective cohort	Taiwan	Total: 43348 GERD: 21674 Non-GERD: 21674	ICD-9 codes 530.11 and 530.81	ICD-9 codes 296.0X, 296.1X, 296.4X, 296.5X, 296.6X, 296.7X, 296.80, atau 296.89	8
You et al (2015) ^16^	Retrospective cohort	Taiwan	Total: 19065 GERD: 3813 Non-GERD: 15252	ICD-9 codes 530.11 atau 530.81	ICD-9 codes 296.0, 296.1, 296.4, 296.5, 296.6, 296.7, 296.8, 296.80, 296.89	9
**Risk of GERD in patients with bipolar disorder**						
Avidan et al (2001) ^18^	Case-control	United States	Total: 216 Bipolar disorder: 18 Non-Bipolar disorder: 198	Clinical symptoms: heartburn, regurgitation, and dysphagia	Diagnostic and Statistical Manual of Mental Disorders	7
Pan et al (2011) ^17^	Retrospective cohort	Taiwan	Total: 80253 Bipolar disorder: 12903 Non-Bipolar disorder: 67350	ICD-9 codes 530.81 atau 530.11	ICD-9 codes 296.0X, 296.1X, 296.4X-296.9X	8

###  Bipolar Disorder in Patients with GERD

**Figure 2:**
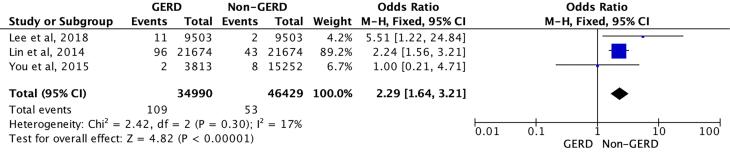
Pooled odds ratios (OR) [95% confidence intervals] for bipolar disorder in patients with GERD compared with non-GERD. The individual study OR is shown as a solid blue box, and the pooled estimate as a solid black diamond. The size of the box is inversely proportional to the standard deviation for the odds ratio. Lines show the 95% confidence intervals

 Of the total of 81 419 patients studied (34 990 patients with GERD and 46 429 patients without GERD), the results showed that there was a positive association with the pooled OR of the occurrence of bipolar disorder in patients with GERD compared with patients without GERD (2.29, 1.64, 3.21, *P* < 0.001) (Figure 2).^14-16^ Subsequently, the range of incidence rates of bipolar disorder (per 1000-person-years) in patients with GERD was 0.13 to 2.16.^14-16^ In addition, the HR of patients with GERD for the development of bipolar disorder compared with patients without GERD was 4.74 (1.05-21.43) at 3 years^15^ and 1.01 (0.21-4.74) at 10 years,^16^ respectively. Subsequently, the incidence rate ratio of bipolar disorder in patients with GERD compared with patients without GERD at 7 years was 2.29 (1.58-3.36).^15^ There were also several independent risk factors for the occurrence of bipolar disorder in patients with GERD, including women (HR 1.78 [1.16–2.74]), age under 60 years (HR 2.35 [1.33–4.16]), and alcohol consumption disorders (HR 4.89 [3.06–7.84]).^14^ Last, patients with longer follow-up duration 1–3 years and ≥ 3 years) were associated with an increased risk of bipolar disorder.^14^

###  GERD in Patients with Bipolar Disorder

**Figure 3:**
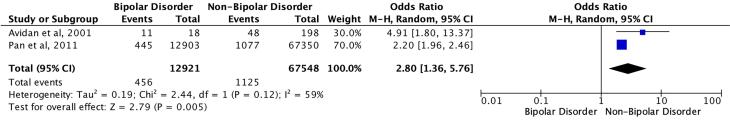
Pooled odds ratios (OR) [95% confidence intervals] for GERD in patients with bipolar disorder compared with non-bipolar disorder. The individual study OR is shown as a solid blue box, and the pooled estimate is shown as a solid black diamond. The size of the box is inversely proportional to the standard deviation for the odds ratio. Lines show the 95% confidence intervals

 Of the total of 80 469 patients studied (12 921 patients with bipolar disorder and 67 458 patients without bipolar disorder), the results showed that there was a positive association with the pooled OR of the occurrence of GERD in patients with bipolar disorder compared with patients with non-bipolar disorder (2.80 [1.36, 5.76]) (*P* = 0.005) (Figure 3).^17,18^ Furthermore, patients with bipolar disorder who received psychoactive drugs were also more at risk of developing GERD than patients with non-bipolar disorder who did not consume psychoactive drugs, with the respective OR being bipolar disorder with zero average daily frequency of drugs was 2.01 [1.77, 2.28], bipolar disorder with one drug was 5.27 [4.18, 6.64], bipolar disorder with two drugs 2.60 [1.75, 3.87], and bipolar disorder with three drugs were 4.75 [2.64, 8.57].^17^ Last, all psychiatric diagnoses, including bipolar disorder, could increase the risk of exercise-induced heartburn as a symptom of GERD with an OR of 3.34 [1.12, 9.96] when compared with non-psychiatric diagnoses.^18^

## Discussion

 The current systematic review and meta-analysis showed evidence of the bidirectional relationship between GERD and bipolar disorder. Patients with GERD have an increased risk of bipolar disorder, and patients with bipolar disorder also have an increased risk of GERD. Previous studies have shown that GERD and bipolar disorder themselves have the same risk factors, namely stress.^22^ Psychosocial stress factors can trigger GERD due to increased perception of intraluminal acid stimulation in the stomach.^22,23^ Subsequently, stress may also cause bipolar disorder, especially in patients who are genetically susceptible to bipolar disorder.^24^

###  Bipolar Disorder in Patients with GERD

 This study showed that there was a positive association with the occurrence of bipolar disorder in patients with GERD. The pathophysiology of GERD itself involves several factors, such as impaired function of the lower esophageal sphincter (LES), the presence of a hiatal hernia, impaired mucosal tolerance to stomach acid content, and impaired esophageal motility.^25^ GERD will stimulate the esophageal mucosa to release large amounts of cytokines, such as interleukin (IL)-6, IL-8, IL-1beta, interferon-gamma (IFN-γ), and tumor necrosis factor-alpha (TNF-α).^26,27^ A large increase in the number of these mediators can cause increased regulation of the inflammatory process in the central nervous system,^28^ which may influence the incidence of bipolar disorder.^29^

 This study also showed that there are several independent risk factors for the occurrence of bipolar disorder in young female patients aged less than 60 years and patients who have a history of alcohol consumption.^14^ Women have a high risk of developing bipolar disorder, possibly due to fluctuations in estrogen levels in women as a trigger factor combined with the presence of a specific variant, namely transglutaminase 2 (TGM2), which has been proven to be essential in the process of apoptosis in the brain. Previous study shows that apoptotic activity in patients diagnosed with bipolar disorder is higher than in patients without bipolar disorders. This allows for the role of estrogen, which can cause anti-inflammatory effects in the brain.^30^ It is possible that an increase in these mediators due to fluctuations in estrogen and GERD can cause increased regulation of the inflammatory process in the central nervous system, thereby increasing the development of bipolar onset in women. In addition, the relationship with alcohol itself is demonstrated by previous studies showing that individuals who use alcohol and bipolar disorder have similar genetic characteristics, neuroimaging results, and also biochemical results.^31^

###  GERD in Patients with Bipolar Disorder

 This study showed that there was a positive association with the occurrence of GERD in patients with bipolar disorder. In general, there are three potential mechanisms by which psychiatric disorders may contribute to the development or exacerbation of GERD, primarily in the poor esophageal motility, including the intrinsic psychological effects, medications consumed, and the indirect impact of a detrimental lifestyle.^18,32^ The increased frequency of reflux symptoms in patients with psychiatric disorders due to impaired esophageal motility may generally be caused by psychiatric medications due to their potential to interfere with esophageal contractile activity.^18^ In certain psychiatric conditions, concomitant esophageal dysfunction can cause acid reflux. Subsequently, high levels of anxiety and affective disorders in patients with psychiatric disorders suggest an association with increased forceful esophageal contractions and non-cardiac chest pain.^33^ In addition to the exacerbation of reflux due to psychological factors, including anxiety and stress, reflux itself can also aggravate stress symptoms, which may increase the likelihood of bipolar disorder events.^24^

 Subsequently, the smoking lifestyle is also more common in patients with psychiatric disorders than in patients without.^34^ Smoking consumption can have a negative impact on the esophageal mucosa. A study conducted by Kahrilas shows that smoking can directly trigger acid reflux and can cause a decrease in LES pressure in the long term.^35^ However, another study by Avidan and colleagues showed the opposite results, where smoking consumption did not have a significant effect on the esophageal mucosa.^18^ Apart from that, this relation could also be because bipolar disorder and GERD have similarities in that the chronic inflammatory process may play a major role in their pathophysiology,^27,28^ and both can be stimulated by stress factors.^23^

 The results of this study also show that patients with bipolar disorder who take psychoactive drugs are also more at risk of developing GERD than patients with non-bipolar disorder who do not take psychoactive drugs. Therapy given to patients with bipolar disorder involves several types of drugs, depending on the phase they are experiencing. Until now, there has been no research that specifically mentions drugs that were used in the mania phase, such as lithium (mood stabilizer), valproate (anticonvulsant), lamotrigine (antiepileptic), and carbamazepine (anticonvulsant) which are related to the incidence of GERD. However, one of the bipolar mania phase medications from the antipsychotic group, namely clozapine, has been proven to increase the incidence of GERD.^36^ Furthermore, antidepressant drugs consumed by patients with bipolar disorder during the depressive phase have also been proven to worsen reflux events.^37^ Tricyclic antidepressant drugs, which have anticholinergic effects, can reduce LES pressure and have been shown to delay gastric emptying, inhibit esophageal motility, and reduce saliva secretion.^36^

 There are also other possible hypotheses, including the impact of sleep and nutrition on patients with bipolar disorder that may influence reflux. Sleep disturbances may occur during all phases of bipolar disorder, with a range of symptoms varying from insomnia, hypersomnia, poor sleep quality, sleep talking, sleepwalking, and obstructive sleep apnea.^38^ These disturbances may lead to dysfunction of the circadian rhythm and abnormalities in the stress axis, inducing hypercortisolemia and increased oxidative stress, which may further lead to pathological conditions such as GERD.^39,40^ In addition, patients with bipolar disorder are associated with an increased risk of weight gain due to disturbances in the energy metabolism pathway and increased appetite caused by the side effects of psychopharmacologic treatments.^41^ Eventually, central obesity can increase abdominal pressure and may aggravate reflux symptoms.^42^

## Strengths and Limitations of Study

**Figure 4:**
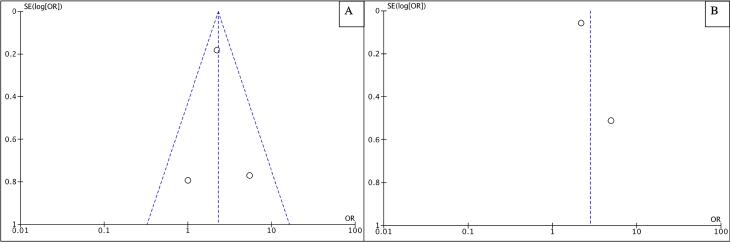
Funnel plot for assessing publication bias. A) Funnel plot of pooled odds ratios (OR) [95% confidence intervals (CI)] for bipolar disorder in patients with GERD compared with non-GERD. B) Funnel plot of pooled OR [95% CI] for GERD in patients with bipolar disorder compared with non-bipolar disorder

 This study has several strengths, including that all studies are of good quality; this can be seen from the quality assessment with NOS, where all studies have a score of more than 6. Subsequently, this study was also conducted on a large sample size and only included longitudinal studies, making it possible to show a clear result regarding the direction of the bidirectional relationship between GERD and bipolar disorder. Furthermore, this study included quantitative parameters in the form of a pooled odds ratio of the bidirectional relationship between GERD and bipolar disorder so that it can strengthen the conclusions drawn. Subsequently, there is the possibility of low publication bias based on symmetry in the funnel plot provided in Figure 4.

 However, this study also has some limitations, including the fact that the studies included in the analysis only come from a few countries, with the majority being Asian countries. Several studies also have weaknesses, including those of You et al and Lee et al, which show significant differences in basic characteristics, especially comorbidities. Subsequently, in You and colleagues’ study, there were also differences in income and degree of urbanization, which may also influence the effect on the occurrence of bipolar disorder. Then, in Avidan and colleagues’ study, the diagnosis of GERD was based only on symptoms of exercise-induced heartburn.

 Last, some parameters were only found in one study, so they may require further investigation, including the HR for the development of bipolar disorder at 3 years and 10 years and the incidence rate ratio at 7 years in patients with GERD. Furthermore, several independent risk factors for bipolar disorder in patients with GERD were only investigated in one study. Then, studies related to increasing the risk of GERD from the number of drugs consumed are also still limited. Therefore, further studies or investigations are recommended to examine several parameters above to obtain more comprehensive results and conclusions.

###  Implications of Study

 Clinicians need to recognize the bidirectional relationship between GERD and bipolar disorder to provide comprehensive care. Regular psychiatric assessments for patients with GERD and screening for GERD in patients with bipolar disorder are essential. Collaboration between gastroenterologists and psychiatrists is crucial for a holistic treatment approach. Individualized treatment plans considering the impact of psychoactive drugs on GERD in patients with bipolar disorder are necessary. Regarding policy, healthcare systems should encourage integrated care models that facilitate collaboration between gastroenterology and mental health services. Policies can support educational initiatives for healthcare professionals to enhance awareness of the bidirectional relationship. Training programs should equip clinicians with the knowledge and skills needed for effective interdisciplinary collaboration.

 Future research should aim to enhance generalizability by examining the diversity of populations. Long-term outcomes, such as the development of bipolar disorder over extended periods, warrant further investigation. In-depth exploration of additional risk factors, especially those identified in only one study, is crucial. Understanding the impact of different medications used in bipolar disorder management on GERD is necessary. Exploring preventive strategies for both GERD in individuals at risk for bipolar disorder and vice versa can contribute to early intervention and prevention programs. The bidirectional association emphasizes the need for a multidisciplinary approach, and addressing these implications can lead to improved outcomes for individuals with these co-occurring conditions.

## Conclusion

 This systematic review and meta-analysis have revealed a bidirectional relationship between GERD and bipolar disorder. The evidence presented underscores the interconnected nature of these conditions, revealing that patients with GERD face an increased risk of developing bipolar disorder and vice versa. This intricate association emphasizes the importance of a comprehensive, multidisciplinary approach to patient care. The findings have significant implications for clinical practice, highlighting the necessity for regular psychiatric assessments in patients with GERD and screening for GERD in individuals with bipolar disorder. Individualized treatment plans, considering the impact of psychoactive drugs, are crucial for optimizing patient outcomes. The identified associations also advocate for integrated care models that bring together gastroenterology and mental health services. In terms of future research, there is a call for studies that encompass diverse populations, investigate long-term outcomes, explore additional risk factors, and delve into the effects of specific medications used in bipolar disorder management on GERD. Last, establishing preventive strategies for both conditions could contribute to early intervention and improved patient care.
